# The Impact of Diet and Betel Nut Use on Skin Lesions Associated with Drinking-Water Arsenic in Pabna, Bangladesh

**DOI:** 10.1289/ehp.7916

**Published:** 2005-09-29

**Authors:** Kathleen M. McCarty, E. Andres Houseman, Quazi Quamruzzaman, Mahmuder Rahman, Golam Mahiuddin, Thomas Smith, Louise Ryan, David C. Christiani

**Affiliations:** 1 Department of Environmental Health, and; 2 Department of Biostatistics, Harvard School of Public Health, Boston, Massachusetts, USA; 3 Dhaka Community Hospital, Dhaka, Bangladesh

**Keywords:** arsenic, Bangladesh, betel nut, case–control, diet

## Abstract

An established exposure–response relationship exists between water arsenic levels and skin lesions. Results of previous studies with limited historical exposure data, and laboratory animal studies suggest that diet may modify arsenic metabolism and toxicity. In this study, we evaluated the effect of diet on the risk of arsenic-related skin lesions in Pabna, Bangladesh. Six hundred cases and 600 controls loosely matched on age and sex were enrolled at Dhaka Community Hospital, Bangladesh, in 2001–2002. Diet, demographic data, and water samples were collected. Water samples were analyzed for arsenic using inductively coupled plasma mass spectroscopy. Betel nut use was associated with a greater risk of skin lesions in a multivariate model [odds ratio (OR) = 1.67; 95% confidence interval (CI), 1.18–2.36]. Modest decreases in risk of skin lesions were associated with fruit intake 1–3 times/month (OR = 0.68; 95%CI, 0.51–0.89) and canned goods at least 1 time/month (OR = 0.41; 95% CI, 0.20–0.86). Bean intake at least 1 time/day (OR = 1.89; 95% CI, 1.11–3.22) was associated with increased odds of skin lesions. Betel nut use appears to be associated with increased risk of developing skin lesions in Bangladesh. Increased intake of fruit and canned goods may be associated with reduced risk of lesions. Increased intake of beans may be associated with an increased risk of skin lesions. The results of this study do not provide clear support for a protective effect of vegetable and overall protein consumption against the development of skin lesions, but a modest benefit cannot be excluded.

From an international perspective, arsenic exposure is one of the most serious environmental health hazards (Gebel 2000). Inorganic arsenic, particularly the trivalent methylated species, is more toxic to human health than the organic form (Chiou et al. 1997; Kitchin 2001). Chronic inorganic arsenic exposure is mainly through drinking water, whereas exposure to the organic form is most commonly through seafood consumption. Drinking-water arsenic exposure is of concern in developing nations where water is not monitored on a regular basis (Gebel 2000).

Arsenic exposure in Bangladesh has been designated a public health emergency by the World Health Organization (Smith et al. 2000). An estimated 25–40 million people have been exposed chronically since the late 1970s (Ahsan et al. 2000). These elevated arsenic concentrations are a result of the mobilization of naturally occurring arsenic from the aquifer to groundwater. The scope of the arsenic problem exceeds any known prior international occurrences (Anawar et al. 2002; Harvey et al. 2002; Nickson et al. 1995).

Premalignant skin lesions, hyperpigmentation, hypopigmentation, and hyperkeratosis are hallmarks of chronic arsenic ingestion by humans (Hughes 2002). Previous studies have found a strong relationship between drinking-water arsenic levels and skin lesions (Guha Mazumder et al. 1998). Skin lesions may be harbingers of increased risk for cancer. After significant exposure, hyperpigmentation develops within 5–15 years, with hyperkeratosis following within a few years (National Research Council 2001). Arsenic-related cancers, such as skin, lung, and bladder cancer, may take decades to develop.

Nutritional deficiencies in diet may increase susceptibility to arsenic-induced skin lesions (Hseuh et al. 1995; Vahter 2000). Previous studies suggest that increased intake of intracellular antioxidants such as selenium and beta carotene may be protective against arsenic toxicity (Hseuh et al. 1995; Styblo and Thomas 2001). The key to the methylation pathway in humans is the transfer of methyl groups by *S*-adenosylmethionine. It has been hypothesized that deficiency in methionine, folate, and vitamin B_12_ could decrease arsenic methylation ability (National Research Council 1999; Vahter 2000). Although metabolism of arsenic by animals is not directly comparable with human arsenic metabolism, animal experiments have shown that protein and methionine intake affect arsenic metabolism efficiency (Kitchin 2001; Maiti and Chatterjee 2001; Vahter and Marafante 1987). No published studies have assessed the potential main effects of diet or modification of arsenic-related skin lesions by diet in Bangladesh.

Previous studies reported that smokers had an increased risk of malignant skin cancers compared with nonsmokers (Erbagci and Erkilic 2002; Zak-Prelich et al. 2004). The association between arsenic-related skin lesions and betel nut and tobacco use has not been assessed. However, head and neck cancers have been associated with betel nut use (Goldenberg et al. 2004; Wu et al. 2004). The International Agency for Research on Cancer (IARC) has classified betel nut quid as a Group 1 carcinogen, regardless if used concurrently with or without tobacco products (IARC 2003). The metabolic pathways of constituents of tobacco and betel nuts are similar: both activate nicotinic receptors and have been associated with appetite suppression (Jo et al. 2002; Strickland et al. 2003).

This investigation was conducted to determine whether diet affects the development of arsenic-related skin lesions. We hypothesized that a higher intake of animal protein, beans, fruits, and vegetables would lower the risk of skin lesions. A secondary hypothesis was that traditional cooking methods using tube-well water may concentrate arsenic in foods such as rice, beans, and vegetables. Intake of these foods may be an additional source of arsenic exposure and may modify the risk of skin lesions. Finally, we sought to determine if betel nut use, smoking, and use of chewing tobacco were associated with increased skin lesions.

## Materials and Methods

### Study population.

This case–control study was conducted in the Pabna District of Bangladesh, located north of Dhaka on the Jamuna River. Pabna was chosen for the following reasons: a range of high and low well-water arsenic levels was suspected due to Pabna’s proximity to the river and prior geologic assessment; Dhaka Community Hospital (DCH) in Dhaka, Bangladesh, has a well-established clinic network in the area; and Pabna is representative of socioeconomic status (SES) of much of nonurban Bangladesh. Eligible cases were residents of Pabna who were at least 16 years of age, with one or more types of skin lesions: diffuse/spotted melanosis, diffuse/spotted keratosis, hyperkeratosis, or leukomelanosis. One control per case was randomly selected from residents of Pabna, loosely matched on age (± 3 years), sex, and geography. Controls were determined to be free of arsenic-related disease. Controls lived in the same village as the case patient but did not share a tube well. One physician, blinded to exposure, made the diagnosis, and treatment was provided at DCH when necessary. Individuals found to have arsenic exposure > 50 μg/L were advised to seek alternative drinking water.

To prevent overmatching on exposure, as in Taiwan (Chen et al. 2003), and to reflect the background exposure distribution, up to 80% of controls were selected from “low-exposure” arsenic (< 50 μg/L) areas, and 20% of the subjects were from “high exposure” (≥ 50 μg/L) areas from within the 52 villages in Pabna. The Bangladesh arsenic drinking-water standard is 50 μg/L. Initial measurements of well arsenic levels were made with Merck Kit for Arsenic Test (sensitive; Merck, Darmstadt, Germany) as described by Kinniburgh and Kosmus (2002). By ensuring heterogeneity of exposure, we were better able to investigate modification of the exposure–response relationship (Greenland 1993). The participation rate was 97.8%; a total of 20 subjects of 920 declined to participate in the study. Reasons for refusal to participate were similar between cases and controls, including fear that giving blood will cause sickness, disbelief that arsenic is a problem, fear of social ramifications of identification as an “arsenic patient,” no desire to participate in any study, and desire for compensation. Informed consent was obtained from all study participants. The study protocol was approved by the institutional review boards at DCH and Harvard School of Public Health.

### Interviews and sample collection.

Trained interviewers administered a questionnaire and collected individual well-water samples. Data were collected on liters of water per liquid per day; frequency of meat, fowl, fish, eggs, bean, rice, bread, canned goods, fruit/juice, vegetable, and milk intake; height; weight; disease history; residential history, including identification of the primary water source (tube well); years of use of water source; use of a previous tube well; and lifestyle factors.

The field team’s collection of water samples was designed to minimize bias. In some cases, field workers may have known if an area was generally high exposure or low exposure. However, the field team did not know the arsenic concentration of the well at the time the subject was examined and interviewed, a procedure similar to a study in West Bengal (Guha Mazumder et al. 1998). It has been documented that wells are often misclassified (Erickson 2003). Thus, the field team was blind to the true exposure level of the subjects when case status was determined. Water samples were analyzed in the United States. The field team received results after subjects were enrolled.

Two drops (0.2 mL) of pure nitric acid was added to each 100-mL water sample upon collection. The samples were stored in a cooler before storage in a 4°C refrigerated room. U.S. Environmental Protection Agency (EPA) method 200.8 (U.S. EPA 1994) with inductively coupled plasma mass spectroscopy (Environmental Laboratory Services, North Syracuse, NY, USA) was used for arsenic analysis. The method limit of detection was 1μg arsenic/L.

### Statistical analysis.

Data were analyzed using SAS (version 8.2; SAS Institute Inc., Cary, NC, USA). We used unconditional logistic regression to calculate crude and adjusted odds ratios (ORs) and 95% confidence intervals (CI), and the loosely matched variables age and sex were included in all models (Rothman and Greenland 1998a, 1998b). To minimize potential for variability in well arsenic level, analysis was restricted to subjects who reported well use for > 6 months. Arsenic level and volume of liquid consumed per day were not combined as a dose variable because liquid volume included juice, milk, soup, tea, and water. Data exploration using generalized additive models (GAMs) in R (version 1.8.1; Free Software Foundation, Inc., Boston, MA, USA) suggested that the log-odds of case identification varied linearly with the arsenic levels of well water. Consequently, untransformed arsenic concentration was treated as a continuous variable in regression models. Intake of food groups was analyzed in individual models. We performed tests for trend across variable categories by including it as a linear term rather than as categorical. Potential confounding factors included frequency of dietary variable intake, smoking status, betel nut use, chewing tobacco, and previous primary tube-well use. To distinguish between the effect of diet, educational status as a marker of SES, and body mass index (BMI), each food group was analyzed four times, controlling for possible combinations of BMI and education. Data exploration using GAMs suggested that the log-odds of case status had a quadratic relationship with BMI. To express potential quadratic effects, two BMI terms were used: BMI centered by subtracting its median (19.1) and the square of the centered BMI. Consolidated categories for education status, age, and frequency of intake of dietary variables were established by combining infrequently observed categories. A protein variable was created for the sum of the frequencies of meat, fish, and fowl intake.

We conducted sensitivity analysis by varying weights of controls selected having a well arsenic concentration < 50 μg/L in a weighted logistic regression analysis. This method was used to determine whether the percentage of controls selected from suspected high- and low-arsenic areas affected the stability of the ORs of all of the covariates in the regression models. The weighting varied between 70 and 95% of controls with suspected low exposure (< 50 μg arsenic/L) and between 30 and 5% of controls with suspected high exposure (≥ 50 μg arsenic/L).

Our sensitivity analysis is based on inverse-probability weighting, which was described succinctly by Zhao et al. (1996). Parameter estimates from a weighted logistic regression estimates are obtained by solving equation:


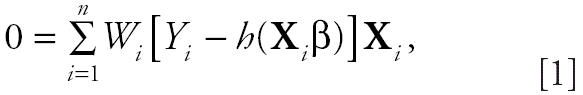


where *i* indexes subject, *Y_i_* is a binary variable representing case status, **X***_i_* is a vector of covariates including arsenic exposure, *h* is the inverse logit function, and *W_i_* is a weight that depends on **X***_i_* and *Y_i_*. The quantity *W_i_*[*Y_i_* *– h*(**X***_i_*β)]**X***_i_* is the weighted score component for subject *i*. The parameter estimates are consistent as long as each weighted score component has zero expectation. This is the case when *W_i_* = 1/π*_i_* , where π*_i_* is the probability of selection into the study (conditional on **X***_i_* and *Y_i_*), as shown by iterated expectation:


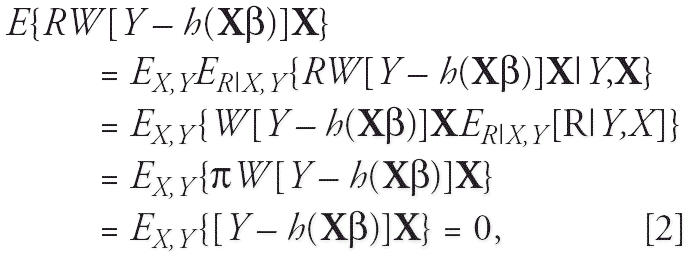


where *R_i_* is a binary random variable indicating selection into the study. When *W_i_* *= C_i_* /π and *C_i_* does not depend on **X***_i_*, Carroll et al. (1995) demonstrate consistency for the non-intercept coefficients.

Thus, when the selection probability π_0_ = *E*(*R_i_* = 1|*Y_i_* = 0) for controls is independent of **X***_i_*, ordinary (unweighted) logistic regression for case–control studies is obtained by setting *C_i_* = 1 for cases and C*_i_* = π_0_ for controls.

If the selection probability for controls depends on **X***_i_* through a dichotomous variable *A_i_*, fully determined by **X***_i_*, the sampling design fixes in advance the probability that *A_i_* = 1. Specifically, the design stipulates that *P*(*A_i_* = 1|*R_i_* = 1, *Y_i_* = 0) = ζ. From an application of Bayes rule,


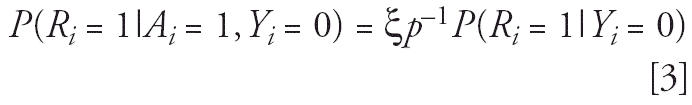


and


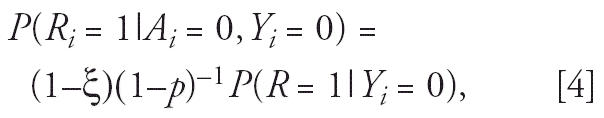


where *p* = *P*(*A_i_* = 1|*Y_i_* = 0).

**Figure 1:**
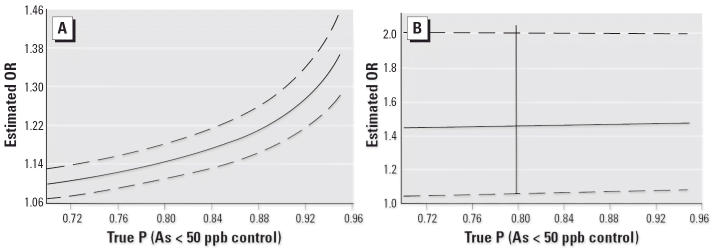
Results from a sensitivity analysis conducted to determine the stability of the main effect of tube-well arsenic concentration in 50 μg/L intervals (*A*) and the stability of the main effect of betel nut use (*B*) on the OR (solid horizontal line) and 95% CI (dashed horizontal lines) for developing skin lesions. The sensitivity analysis evaluated the influence of control selection allowing for 70–95% of the controls being selected from tube wells containing < 50 μg/L. The vertical line represents the sampling design employed in this study, which assumed that the 80% of the tube wells in Pabna contained arsenic concentrations < 50 μg/L. As the percentage of controls with arsenic concentrations < 50 μg/L increases, the OR for skin lesions increases associated with each 50-μg/L increase in tube-well arsenic. The *x*-axis is the percentage of controls selected from areas suspected to have well-water arsenic concentration < 50 μg/L. ORs and 95% CIs show the stability of the effect estimates as the percentage of controls from low-exposure areas are varied in the logistic regression model.

When the stipulated distribution matches the true distribution, *p* = ζ, it is clear that unweighted logistic regression produces consistent estimates, as shown by setting *C_i_* = *P*(*R_i_* = 1|*Y_i_* = 0). Sensitivity of parameter estimates to the true underlying distribution of *A_i_* can be assessed by varying the parameter *p* among plausible values. For each value of *p*, weighted logistic parameter estimates are obtained by using weights ζ*p*^–1^ for controls with *A_i_* = 1, (1– ζ)(1–*p*)^–1^ for controls with *A_i_* = 0, and unit weights for cases. Results can be examined graphically in Figure 1.

We investigated effect modification with arsenic for any type of food that is cooked in water or could potentially be prepared with water (e.g., dry milk); interaction terms were not included for meat, fowl, fish, bread, and eggs. Again, each model was analyzed four times to investigate the effect of diet, SES, and BMI. Additionally, potential main effects and modifying effects of diet were investigated in the low-arsenic exposure group.

## Results

The 596 cases of skin lesions included 73 spotted keratosis cases, 117 diffuse keratosis cases, 145 spotted melanosis cases, 377 diffuse melanosis cases, 40 hyperkeratosis cases, and 342 leukomelanosis cases. Some individuals had multiple lesion types. Cases had significantly higher well arsenic concentrations compared with control subjects (Table 1). Controls reported significantly higher previous tube-well use, shorter duration of current tube-well use, and higher educational status than cases. Frequencies of fruit/juice intake and bread intake were significantly different between cases and controls (Table 2).

Betel nut users had an increased risk of skin lesions (OR = 1.67; 95% CI, 1.18–2.36) (Table 3). Smoking and use of chewing tobacco were not significantly related to skin lesions. All dietary models were adjusted for smoking status and use of betel nut and chewing tobacco.

There was a strong exposure–response relationship between arsenic level of tube-well water and skin lesions. In the multivariate adjusted model, there was a 1.14 (95% CI, 1.10–1.17) log odds increase in skin lesions for every 50-μg/L increase of arsenic in tube-well water. There was no significant relationship between liters of fluid consumed per day and case status. Sensitivity analysis of estimates for skin lesion risk predicted by well arsenic concentration varied with the weighting of controls selected from suspected high- and low-arsenic areas. Increasing the percentage of controls with drinking-water As exposure < 50 μg/L did not overestimate the risk of skin lesions. The increased risk of skin lesions with increasing arsenic exposure remained statistically significant (Figure 1), and selection of controls did not bias the results for the other dietary and lifestyle variables.

Of the dietary intake models, intake of fruit and canned goods was associated with reduced risk of skin lesions. Bean consumption was associated with an increased risk of lesions (Table 4). Each multivariate model adjusted for age, sex, previous well use, well arsenic concentration, daily liquid intake, smoking status, chewing tobacco, betel nut use, and the four possible combinations of BMI and education. Fruit intake 1–3 times/month was associated with a reduced risk of skin lesions (OR = 0.68; 95% CI, 0.51–0.89) compared with fruit intake < 1 time/month. Fruit intake > 3 times/month was not significantly associated with skin lesions. Bread intake 1–3 times/month was associated with an increased risk of skin lesions compared with intake < 1 time/month in the crude model only. Bean intake at least once per day was associated with almost twice the risk of skin lesions compared with less than once per month (OR = 1.89; 95% CI, 1.11–3.22). Rice, vegetables, eggs, fish, fowl, milk, and beef intake were not significantly associated with skin lesions. There was no evidence of significant interactions between arsenic level of well water and foods prepared with well water in the cooking process. When the analysis was restricted to subjects with well arsenic levels < 50 μg/L, the results were similar to those presented in Table 4.

## Discussion

Results were consistent with previous studies in showing that the concentration of arsenic in tube wells increased the risk of skin lesions (Guha Mazumder et al. 1998; Tondel et al. 1999); however, because of the sampling, the effect estimate may be biased. Sensitivity analysis indicated that estimates for odds of skin lesions associated with each 50 μg/L increase in well arsenic concentration varied slightly with the proportion of controls selected with As exposure < 50 μg/L. There was a potential bias because of control selection based on exposure; increasing the percentage of controls with exposure < 50 μg/L results in an overestimation of risk of skin lesions. However, the increased risk of skin lesions with increasing arsenic exposure remained statistically significant (Figure 1). Our selection distribution of controls, 84.5% of wells with < 50 μg/L arsenic and 15.5% of wells with > 50 μg/L arsenic, was consistent with the known background arsenic exposure distribution of Pabna tube wells conducted by the British Geological Survey (BGS): 81.2% of wells with < 50 μg/L arsenic and 18.8% of wells with > 50 μg/L arsenic [BGS and Bangladesh Department of Public Health Engineering (DPHE) 2001]. The control selection was representative of the background exposure distribution of Pabna. There was a potential for selection bias if the potential controls based on age and sex did not have the same distribution of wells above and below 50 μg/L arsenic as the general population. Results for effect estimates for dietary variables, betel nut use, or other nonarsenic-related predictors were stable over the varying exposure assumptions (Figure 1).

Our results indicate that betel nut use increases the risk of skin lesions. This practice has been associated with head and neck cancers in Bangladesh and elsewhere (Carr 1986). Strickland and colleagues (Strickland and Duffield 1997; Strickland et al. 2003), reported that betel nut use differentially altered fat and protein metabolism, that carbohydrate metabolism was higher in users compared with nonusers, and that hunger was suppressed after betel nut use. Chewing betel nuts has been a practice used to suppress hunger in India (Krishnamurthy 1997). Based on our data, BMI was not significantly different between betel nut users and non-users (*p* = 0.10), and there was no correlation in our data between betel nut use and BMI (correlation = 0.06, *p* = 0.09). Whether risk is confounded by the constituents of betel nuts or through another mechanism remains unclear from our results and was beyond the scope of this study. Our results do not support effect modification of skin lesions by betel nut use and arsenic concentration of drinking water (*p* = 0.07). This finding poses implications for further research.

BMI was not significantly related to risk of developing skin lesions. Findings on BMI and skin lesions from West Bengal varied based on arsenic exposure level (Guha Mazumder et al. 1998; Haque et al. 2003).

Modification of the relationship between arsenic exposure and skin lesions by increased intake of animal protein as originally hypothesized was not detected. Frequency of fowl, fish, beef, and egg as protein sources was not statistically significant in any of the final models. Laboratory animal studies of arsenic metabolism and protein intake conflict; however, arsenic metabolism is different in humans than animals in terms of methylation and health outcomes (Kitchin 2001; Maiti and Chatterjee 2001; Vahter and Marafante 1987).

Increased bean intake was significantly associated with skin lesions. The arsenic concentration in cooked food has been found to be dependent on the arsenic level of the water used for cooking, the volume of water used, and the length of cooking time (Bae et al. 2002; Del Razo et al. 2002). Beans contain hemicellulose, which retains water after the cooking process and possibly concentrates inorganic arsenic, as well (Diaz et al. 2004). We did not detect effect modification by bean intake on the association between well arsenic concentration and skin lesions (*p* = 0.23); however, we may not have had adequate power to detect this association. It is possible that arsenic concentrates in the dishes containing beans and that this serves as a secondary source of arsenic exposure, but further studies measuring the arsenic levels of food prepared in Bangladesh using traditional cooking methods are needed.

Similarly to beans, rice and vegetables are boiled with excessive amounts of water for an extended duration (Bae et al. 2002; Jahan and Hossain 1998). A study in Bangladesh concluded that the method of cooking and arsenic level in water used does affect the amount of arsenic in cooked rice, suggesting a chelating effect by the rice or concentration of arsenic due to the evaporation of water during the cooking process (Bae et al. 2002). Rice was not a significant modifier, but with 87.8% of the subjects reporting rice consumption > 3 times/day, 10.7% of subjects reporting rice intake 1–2 times/day, and 1.5% of subjects reporting rice intake < 1 time/day, we may not have had the power to detect any significant association. Our study did not find significant main effects or effect modification by rice or vegetables.

Arsenic may be integrated into fruit and vegetables through high-arsenic irrigation, although results of previous studies indicate that this is an unlikely source of significant arsenic exposure. Arsenic is not easily incorporated into plants (Del Razo et al. 2002). Sancha et al. (1992) noted that vegetables grown in areas of high-arsenic irrigation had higher arsenic in their peels but not in the edible portion of the raw vegetable. Arsenic concentration of fruits and vegetables depends on which portion is consumed (Carbonell-Barrachina et al. 1997). Studies have generally found that in plants, the arsenic concentration is greatest in the roots of plants, then stems and leaves, and then fruit and seeds (Carbonell-Barrachina et al. 1997; Rosas et al. 1999; Van den Broeck et al. 1998). We did not determine which types of vegetables were consumed from our study. Further studies are needed to measure the arsenic levels in cooked and raw vegetables in Bangladesh.

Increased frequency of fruit intake was found to be associated with reduced risk of arsenic-related skin lesions (Table 4). Certain fruits, such as mangos (*aam*) and red pumpkin (*mishti kumra*), which are prevalent in the Bangladeshi diet, are high in carotenoids and other nutrients. Hseuh et al. (1995) reported that skin cancer cases had significantly lower serum beta carotene levels compared with controls. Because fruit is generally consumed raw, or quick-fried in oil, it does not accumulate arsenic through traditional cooking methods, and the flesh of the fruit has the lowest arsenic concentration (Rosas et al. 1999; Vahter and Marafante 1987). The intake of canned goods was also associated with a decreased risk of skin lesions; however, it is unclear what type of foods were consumed. The interpretation of this finding is difficult and is limited by the number of subjects.

Milk consumption was not shown to have a main effect or to modify risk of arsenic-associated skin lesions. Study results conflict regarding whether arsenic is transferred through cow’s milk at a significant level (Saha et al. 1999; Sekhar et al. 2003; Stevens 1991). Arsenic levels in milk remain an area of future study.

We acknowledge several limitations to our study. With one water sample per subject, we assumed no significant temporal variability in arsenic concentration. Results of previous studies indicate that there was little variability in well arsenic concentration over time (Dhar et al. 2003; Van Geen et al. 2002); however, we recognize this limitation. Because there may be some variability in wells < 6 months old, we excluded those wells from our analysis. One sample taken from the home may not represent the arsenic level of water consumed outside of the home. It was likely that any bias introduced by this exposure misclassification was nondifferential. Furthermore, this population was known to not move outside of the village, and well use is stable. Information bias was possible if people with high-arsenic wells were more aware of arsenic levels in their drinking water and were more likely to come for treatment than people living in areas thought to have low arsenic levels. Recall bias related to diet may have existed if the subject had some knowledge regarding the role of nutrition in the arsenic–skin lesion relationship, resulting in differential misclassification and biasing results away from the null. However, given the educational status of the population and the lack of concrete evidence related to diet and arsenic metabolism, this bias is unlikely. The use of a food frequency questionnaire did not make it possible to analyze for specific micronutrients because there were no serving size estimates, nor were specific types of foods identified. Moreover, the food frequency questionnaire was not validated in the population before its use. Despite these limitations, we detected significant differences in risk of developing skin lesions based on the report of frequency of intake of fruit, beans, and canned goods.

Our study has several strengths. Measures were taken to ensure team uniformity in obtaining information from subjects. The field team could not have known the level of the potential subject’s true arsenic exposure during subject recruitment. Diagnostic criteria used to identify skin lesions were developed in this region of the world, and the physician was well trained and routinely diagnoses these lesions. Results of the sensitivity analysis indicated that the main effects of fruit, canned goods, and bean intake as well as significant non-arsenic-related variables, such as betel nuts, were stable irrespective of the proportion of controls selected with arsenic levels < 50 μg/L in well water.

Although there have been several studies in other regions of the world that measured arsenic exposure through the diet, there are currently no published epidemiologic studies of skin lesion risk modified by diet in Bangladesh (Alam et al. 2003; Haque et al. 2003; Mitra et al. 2004; Queirolo et al. 2000). In conclusion, betel nut use was consistently associated with an increased risk of skin lesions. This is the first published study to associate betel nut use with an increased risk of skin lesions. Betel nut use may be a potential effect modifier of arsenic-related skin lesions, although our results do not support effect modification. The results of this study do not provide clear support for a protective effect of vegetable and overall protein consumption against the development of skin lesions, but a modest benefit cannot be excluded. Our results suggest a benefit of increased fruit intake and a potential increased risk associated with bean intake. Uncertainties about the arsenic content in food remain, and additional studies are needed to determine the bioavailability of arsenic from food (Del Razo et al. 2002).

## Correction

The authors found several errors in the original manuscript published online:

- Figure 1A was incorrect; it has been corrected here.
- Instead of being “loosely matched,” cases and controls were frequency matched on age and sex.
- The authors would like to clarify that this study was designed not to investigate the main effects of arsenic exposure on skin lesion but to investigate modifiers of this relationship.
- In the “Results” and the “Discussion,” respectively, the authors state that “There was a strong exposure–response relationship between arsenic level of tube-well water and skin lesions” and “the increased risk of skin lesions with increasing arsenic exposure remained statistically significant.” However, they actually could not interpret the main effects of arsenic on skin lesions because of control selection, as shown by the sensitivity analysis (Figure 1).

## Figures and Tables

**Table 1:** Characteristics of skin-lesion cases and population-based controls in Pabna, Bangladesh (mean ± SD, except where noted).

	Controls (*n* = 596)	Cases (*n* = 593)	*p*-Value
Age (years)	33.7 ± 12.6	33.9 ± 12.7	0.98
BMI (kg/m^2^)	20.4 ± 3.1	20.1 ± 3.1	0.53
Male [*n* (%)]	360 (60.4)	357 (60.2)	0.94
Duration of present well use (years)	10.2 ± 9.0	8.0 ± 7.2	0.004
Reported a previous well [*n* (%)]	17 (2.9)	46 (7.8)	0.002
As level of current well (μg/L)	66.2 ± 149.6	232.8 ± 315.72	< 0.0001
Daily total water/liquid consumption (L)	3.8 ± 1.2	3.7 ± 1.1	0.63
Ever used betel nuts [*n* (%)]	145 (24.3)	164 (27.7)	0.19
Years of betel nut use	10.8 ± 8.9	11.0 ± 9.5	0.69
Chew tobacco leaves [*n* (%)]	96 (16.1)	101 (17.0)	0.06
Years of tobacco leaves chewed	9.9 ± 9.1	10.9 ± 9.4	0.40
Smokes cigarettes currently [*n* (%)]	182 (30.5)	158 (26.6)	0.14
Ever smoked [*n* (%)]	185 (31.0)	170 (28.7)	0.37
Education level [*n* (%)]			0.002
Illiterate	104 (17.5)	136 (32.9)	
Literate (incomplete primary education)	142 (23.8)	174 (29.3)	
Completed primary education	78 (13.1)	71 (12.0)	
Completed middle school education	191 (32.1)	139 (23.4)	
Completed secondary education or more	81 (13.6)	73 (12.3)	

**Table 2:** Frequency of consumption of dietary variables.

Intake	Cases [% (no.)]	Controls [% (no.)]	*p*-Value
Fruit/juice (*n* = 747)			0.02
< 1 time/month (*n* = 181)	28.7 (105)	20.0 (76)	
1–3 times/month (*n* = 478)	59.8 (219)	68.0 (259)	
> 3 times/month (*n* = 88)	11.5 (42)	12.0 (46)	
Beef (*n* = 1,155)			0.11
< 1 time/month (*n* = 114)	9.4 (54)	10.3 (60)	
1–3 times/month (*n* = 748)	67.7 (389)	61.9 (359)	
> 3 times/month (*n* = 293)	23.0 (132)	27.8 (161)	
Canned goods (*n* = 232)			0.19
< 1 time/week (*n* = 141)	66.7 (80)	54.5 (61)	
1–6 times/week (*n* = 40)	16.7 (20)	17.9 (20)	
> 6 times/week (*n* = 51)	16.7 (20)	27.7 (31)	
Bread (*n* = 993)			0.03
< 1 time/month (*n* = 692)	69.8 (346)	69.6 (346)	
1–3 times/month (*n* = 74)	9.5 (47)	5.4 (27)	
> 3 times/month (*n* = 227)	20.8 (103)	25.0 (124)	
Milk (*n* = 1,024)			0.82
< 1 time/month (*n* = 240)	23.8 (125)	21.3 (115)	
1–3 times/month (*n* = 325)	30.9 (162)	32.7 (163)	
> 1 time/week (*n* = 459)	45.3 (238)	44.3 (221)	
Beans (*n* = 1,053)			0.81
≤ 3 time/month (*n* = 813)	77.8 (407)	76.6 (406)	
1–6 times/week (*n* = 59)	5.7 (30)	5.5 (29)	
> 6 times/week (*n* = 181)	16.4 (86)	17.9 (95)	
Fowl (*n* = 1,115)			0.58
< 1 time/month (*n* = 184)	16.8 (93)	16.3 (91)	
1–3 times/month (*n* = 743)	67.9 (377)	65.4 (366)	
> 3 times/month (*n* = 188)	15.3 (85)	18.4 (103)	
Fish (*n* = 1,171)			0.32
< 1 time/week (*n* = 48)	3.8 (22)	4.4 (26)	
1–6 times/week (*n* = 962)	83.9 (488)	80.5 (474)	
> 6 times/week (*n* = 161)	12.4 (72)	15.1 (89)	
Eggs (*n* = 1,121)			0.21
< 1 time/week (*n* = 536)	49.6 (273)	46.0 (263)	
1–6 times/week (*n* = 550)	48.7 (268)	49.4 (282)	
> 6 times/week (*n* = 35)	1.6 (9)	4.6 (26)	
Vegetables (*n* = 1,157)			0.65
< 1 time/week (*n* = 40)	3.4 (20)	3.5 (20)	
1–6 times/week (*n* = 718)	63.3 (368)	60.8 (350)	
> 6 times/week (*n* = 399)	33.2 (193)	35.8 (206)	
Rice (*n* = 1,179)			0.67
< 1 time/day (*n* = 15)	1.0 (6)	1.5 (9)	
1–2 times/day (*n* = 188)	16.0 (94)	15.9 (94)	
> 2 times/day (*n* = 976)	82.9 (486)	82.6 (490)	

**Table 3:** Crude and adjusted ORs and 95% CIs for nondietary variables.

	Cases	Controls	Adjusted OR^a^ (95% CI)
Educational status	593	596	
Illiterate	136	104	1.0
Literate (incomplete primary education)	174	142	0.96 (0.66–1.40)
Completed primary education	71	78	0.76 (0.47–1.22)
Completed middle school education	139	191	0.62 (0.41–0.94)
Completed secondary education or more	73	81	0.78 (0.47–1.30)
Well-water As (*n* = 1,189)	593	596	
As (per 50 μg/L)	593	596	1.14 (1.10–1.17)
Liquid/day (L)	593	596	0.93 (0.83–1.05)
Age (per 10-year increase)	593	596	0.98 (0.87–1.11)
Sex			
Males	357	360	0.83 (0.60–1.14)
Females	236	236	
Previous well use (*n* = 1,189)	46	17	4.02 (2.10–7.70)
BMI (*n* = 1,189)	593	596	
Median			0.96 (0.90–1.01)
Median^2^			1.01 (1.00–1.01)
Betel nut use (*n* = 1,189)	164	145	1.67 (1.18–2.36)
Chewing tobacco (*n* = 1,189)	101	96	0.84 (0.70–1.01)
Cigarette use (*n* = 1,189)	158	182	0.86 (0.61–1.21)

a Adjusted for well arsenic concentration, daily total liquid intake age, BMI, educational status (SES), previous well use, sex, chewing tobacco use, and betel nut use.

**Table 4:** ORs (95% CIs) for the effect of dietary intake on case status.

	Crude	Adjusted^a^	Adjusted^b^	Adjusted^c^	Adjusted^d^
Fruit
< 1 time/month^e^	1.0	1.0	1.0	1.0	1.0
1–3 times/month	0.65 (0.50–0.83)	0.66 (0.50–0.86)	0.66 (0.50–0.87)	0.67 (0.51–0.89)	0.68 (0.51–0.89)
> 3 times/month	0.93 (0.59–1.46)	0.98 (0.59–1.63)	0.98 (0.59–1.63)	1.02 (0.61–1.71)	1.03 (0.62–1.73)
Trend	0.09	0.19	0.21	0.24	0.23
Beef
< 1 time/month^e^	1.0	1.0	1.0	1.0	1.0
1–3 times/month	1.03 (0.72–1.47)	1.16 (0.77–1.75)	1.21 (0.80–1.84)	1.23 (0.81–1.87)	1.19 (0.80–1.80)
> 3 times/month	0.70 (0.46–1.04)	1.01 (0.63–1.62)	1.07 (0.67–1.72)	1.15 (0.71–1.86)	1.11 (0.69–1.80)
Trend	0.03	0.84	0.98	0.71	0.76
Canned goods
< 1 time/month^e^	1.0	1.0	1.0	1.0	1.0
1–6 times/week	0.99 (0.52–1.88)	0.86 (0.42–1.78)	0.88 (0.43–1.82)	0.85 (0.41–1.76)	0.89 (0.41–1.72)
> 6 times/week	0.46 (0.24–0.88)	0.41 (0.20–0.85)	0.43 (0.21–0.88)	0.43 (0.21–0.89)	0.41 (0.20–0.86)
Trend	0.008	0.01	0.02	0.02	0.01
Bread
< 1 time/month^e^	1.0	1.0	1.0	1.0	1.0
1–3 times/month	2.35 (1.45–3.82)	1.62 (0.92–2.86)	1.63 (0.92–2.88)	1.65 (0.93–2.93)	1.65 (0.93–2.92)
> 3 times/month	0.96 (0.71–1.30)	0.95 (0.68–1.34)	0.96 (0.68–1.35)	1.04 (0.73–1.48)	1.04 (0.74–1.48)
Trend	0.82	0.64	0.67	0.98	0.98
Milk
< 1 time/month^e^	1.0	1.0	1.0	1.0	1.0
1–3 times/month	1.11 (0.84–1.45)	1.12 (0.83–1.50)	1.16 (0.86–1.57)	1.14 (0.84–1.54)	1.16 (0.86–1.57)
> 3 times/month	1.03 (0.77–1.41)	1.17 (0.84–1.64)	1.20 (0.86–1.68)	1.18 (0.84–1.65)	1.20 (0.86–1.68)
Trend	0.59	0.46	0.58	0.59	0.98
Beans
≤ 3 times/month^e^	1.0	1.0	1.0	1.0	1.0
1–6 times/week	0.52 (0.32–0.85)	0.64 (0.38–1.08)	0.65 (0.38–1.10)	0.68 (0.40–1.16)	0.69 (0.40–1.17)
> 6 times/week	1.55 (1.01–2.38)	1.78 (1.06–3.00)	1.80 (1.07–3.04)	1.86 (1.10–3.16)	1.89 (1.11–3.22)
Trend	0.96	0.87	0.84	0.62	0.66
Fowl
< 1 time/month^e^	1.0	1.0	1.0	1.0	1.0
1–3 times/month	1.05 (0.79–1.41)	1.04 (0.76–1.44)	1.05 (0.77–1.45)	1.11 (0.80–1.54)	1.11 (0.80–1.54)
> 3 times/month	0.72 (0.49–1.07)	0.95 (0.62–1.46)	0.98 (0.63–1.51)	1.09 (0.70–1.71)	1.09 (0.70–1.70)
Trend	0.32	0.85	0.73	0.43	0.46
Fish
< 1 time/week^e^	1.0	1.0	1.0	1.0	1.0
1–6 times/week	0.84 (0.51–1.38)	0.86 (0.49–1.51)	0.85 (0.48–1.50)	0.86 (0.49–1.53)	0.86 (0.49–1.51)
> 6 times/week	0.76 (0.42–1.36)	0.72 (0.37–1.40)	0.72 (0.37–1.40)	0.75 (0.39–1.46)	0.75 (0.39–1.46)
Trend	0.34	0.21	0.23	0.29	0.29
Eggs
< 1 time/week^e^	1.0	1.0	1.0	1.0	1.0
1–6 times/week	0.91 (0.72–1.15)	0.98 (0.76–1.27)	0.97 (0.75–1.26)	0.99 (0.76–1.29)	0.98 (0.76–1.28)
> 6 times/week	0.50 (0.23–1.08)	0.60 (0.26–1.38)	0.60 (0.26–1.37)	0.68 (0.30–1.57)	0.67 (0.29–1.54)
Trend	0.67	0.67	0.62	0.72	0.69
Vegetables
< 1 time/day^e^	1.0	1.0	1.0	1.0	1.0
1–2 times/day	0.91 (0.72–1.15)	0.91 (0.69–1.21)	0.91 (0.68–1.20)	0.93 (0.70–1.24)	0.93 (0.70–1.24)
> 2 times/day	0.50 (0.23–1.08)	0.96 (0.40–2.29)	1.0 (0.42–2.37)	0.99 (0.41–2.36)	1.02 (0.43–2.45)
Trend	0.67	0.24	0.25	0.26	0.28
Rice
< 1 time/day^e^	1.0	1.0	1.0	1.0	1.0
1–2 times/day	0.86 (0.37–2.00)	1.0 (0.39–2.52)	1.01 (0.40–2.56)	1.09 (0.43–2.75)	1.09 (0.43–2.75)
> 2 times/day	0.82 (0.37–1.83)	0.86 (0.36–2.07)	0.85 (0.35–2.06)	0.86 (0.36–2.06)	0.85 (0.36–2.06)
Trend	0.94	0.80	0.81	0.81	0.82
Protein
Low	1.0	1.0	1.0	1.0	1.0
Medium	0.64 (0.44–0.95)	0.76 (0.50–1.15)	0.77 (0.51–1.17)	0.81 (0.53–1.24)	0.81 (0.53–1.24)
High	0.73 (0.55–0.97)	0.84 (0.61–1.15)	0.85 (0.62–1.17)	0.90 (0.65–1.24)	0.90 (0.65–1.25)
Trend	0.01	0.17	0.21	0.34	0.36

a Adjusted for age, sex, previous well use, well arsenic concentration, daily total liquid intake, smoking status, chewing tobacco use, and betel nut use.

b Adjusted for age, sex, previous well use, well arsenic concentration, daily total liquid intake, smoking status, chewing tobacco use, betel nut use, and BMI.

c Adjusted for age, sex, previous well use, well arsenic concentration, daily total liquid intake, smoking status, chewing tobacco use, betel nut use, and SES (education).

d Adjusted for age, sex, previous well use, well arsenic concentration, daily total liquid intake, smoking status, chewing tobacco use, betel nut use, BMI, and SES (education).

e Reference category.
